# *RPN2* Gene Confers Osteosarcoma Cell Malignant Phenotypes and Determines Clinical Prognosis

**DOI:** 10.1038/mtna.2014.35

**Published:** 2014-09-02

**Authors:** Tomohiro Fujiwara, Ryou-u Takahashi, Nobuyoshi Kosaka, Yutaka Nezu, Akira Kawai, Toshifumi Ozaki, Takahiro Ochiya

**Affiliations:** 1 Division of Molecular and Cellular Medicine, National Cancer Center Research Institute, Tokyo, Japan; 2 Division of Musculoskeletal Oncology, National Cancer Center Hospital, Tokyo, Japan; 3 Department of Orthopaedic Surgery, Okayama University Graduate School of Medicine, Dentistry, and Pharmaceutical Sciences, Okayama, Japan

**Keywords:** drug response, metastasis, osteosarcoma, ribophorin II (RPN2), RNA interference

## Abstract

Drug resistance and metastasis are lethal characteristics of tumors. We previously demonstrated that silencing of ribophorin II (RPN2), which is part of the N-oligosaccharyl transferase complex, efficiently induced apoptosis and reduced resistance to docetaxel in human breast cancer cells. Here, we report the clinical and functional correlations of RPN2 expression in osteosarcoma. Immunohistochemical evaluation of 35 osteosarcoma patient biopsies revealed that RPN2 was moderately to highly expressed in all specimens, and higher RPN2 mRNA expression was significantly correlated with poor prognosis. To investigate whether lethal phenotypes of osteosarcoma could be reduced by regulating the expression of RPN2, we conducted a study of RNAi-induced RPN2 knockdown in highly metastatic human osteosarcoma cells. The results indicated that RPN2 silencing reduced cell proliferation, sphere formation, cell invasion, and sensitized drug response *in vitro*. Mice bearing RPN2-silenced highly metastatic osteosarcoma xenografts showed reduced tumor growth and lung metastasis, and survived longer than mice bearing control tumor xenografts. Taken together, our data suggest that RPN2 silencing contributes to regulation of lethal osteosarcoma phenotypes and could be a novel target for RNAi-based therapeutics against osteosarcoma.

## Introduction

The most lethal characteristics of tumors include drug resistance and metastasis.^1,2,3,4,5^ Osteosarcoma is no exception, and various cohort studies have shown that both response to chemotherapy and metastasis are independent prognostic factors.^6,7,8,9,10,11,12,13^ Osteosarcoma is the most common primary bone malignancy arising in children and young adults.^6,14,15^ Along with the development of multi-agent chemotherapy and surgical techniques including the concepts of surgical margins and reconstruction,^16,17^ patient prognosis has gradually improved over the past 30 years. Current chemotherapeutic regimens including pre- and postoperative doxorubicin, cisplatin, methotrexate, and/or ifosfamide have maintained 5-year overall survival rates at approximately 60–80%.^11,12^ However, osteosarcoma patients who show a poor response to chemotherapy or who have multiple pulmonary metastases have a poor prognosis, with an overall survival rate of <50% and <30%, respectively.^10,18^ The molecular background supports these data, as the presence of increased levels of P-glycoprotein^9^ or metastasis-related genes such as ezrin^19^ in tumor cells has been associated with a significantly poor prognosis of osteosarcoma patients. Therefore, the development of a novel approach targeting these key molecules would provide new hope for patients.

Our previous study showed that downregulation of ribophorin II (RPN2), which is part of the N-oligosaccharyl transferase complex, efficiently induced apoptosis in docetaxel-resistant human breast cancer cells in the presence of docetaxel.^20^ Silencing of RPN2 decreased membrane localization of P-glycoprotein through a reduction of glycosylation status, and restored sensitivity to docetaxel. These results indicated that regulation of RPN2 expression contributes to a more effective response to docetaxel-based chemotherapy. However, it has been unclear whether these mechanisms would be effective in other cancers, including neoplasms of mesenchymal origin. In this study, we examined RPN2 expression using immunohistochemical staining and quantitative real-time polymerase chain reaction (qRT-PCR) of pretreatment biopsy samples from patients with osteosarcoma, and evaluated the correlation between RPN2 expression and clinicopathological features. In addition, we investigated whether the level of RPN2 expression affected cell proliferation, drug sensitivity, sphere formation ability, and cell invasion in osteosarcoma *in vitro*, as well as tumor growth and metastatic ability *in vivo*.

## Results

### High expression of RPN2 in osteosarcoma biopsies is significantly correlated with poor patient survival

**Figure 1:**
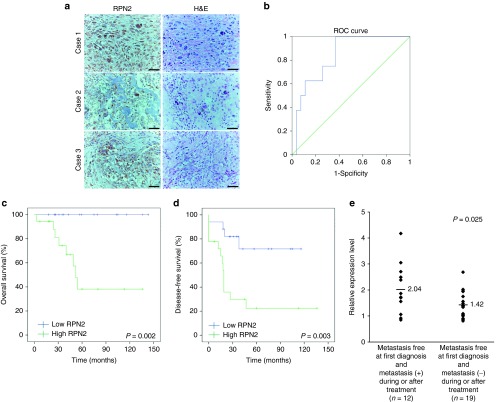
**Clinical relevance of RPN2 expression in osteosarcoma.** (**a**) Immunohistochemical staining of RPN2 protein and hematoxylin and eosin staining in osteosarcoma biopsy specimens of osteosarcoma. RPN2 protein expression was moderately to strongly detected in the cytoplasm in all biopsy samples. Scale bar, 50 μm. (**b**) ROC curve for expression of RPN2. The area under the ROC curve was 0.838. The cutoff was set at the point representing 100% sensitivity and 63.0% specificity. (**c**) The Kaplan–Meier curves for overall survival according to the RPN2 expression (log-rank test; *P* = 0.002). (**d**) The Kaplan–Meier curves for disease-free survival according to the RPN2 expression (log-rank test; *P* = 0.003). (**e**) RPN2 expression in biopsy specimens of primary osteosarcoma. Thirty-one specimens of primary osteosarcoma were divided into two groups: cases remaining metastasis-positive during or after treatment (*n* = 12, left) and cases remaining metastasis-free for at least 3 years after treatment (*n* = 19, right). The average value for each dataset is shown as a horizontal line. *P* values were calculated using Welch's *t*-test (*P* = 0.025).

We evaluated tissue samples from 35 osteosarcomas obtained by diagnostic incisional biopsy of primary osteosarcoma at the National Cancer Center Hospital, Japan, between 1997 and 2010. Immunohistochemically, RPN2 protein was moderately to strongly expressed in all of these specimens, and localized in the cytoplasm (**Figure 1a**). RPN2 protein expression was negative to weakly positive in normal tissues, including mesenchymal tissues such as adipose or fibrous tissues, which was consistent with the findings of our previous study.^20^ We next performed qRT-PCR using cDNA obtained from these osteosarcoma patients and evaluated the clinicopathological features according to the expression of RPN2 in the same cohort set. We determined the cutoff point that yielded optimum sensitivity and specificity using receiver-operating characteristic (ROC) curve analysis. The area under the ROC curve was 0.838 (**Figure 1b**), and Kaplan–Meier analysis showed that high levels of RPN2 expression were associated with significantly worse overall survival rates (log-rank test, *P* = 0.002; **Figure 1c**) and disease-free survival rates (log-rank test, *P* = 0.003; **Figure 1d**). In this statistical analysis, all low-RPN2 patients survived, indicating that the expression of RPN2 had significant prognostic value. The clinicopathological features of the patients in relation to the expression of RPN2 are summarized in **Table 1**. Univariate analysis revealed a significant correlation between high-RPN2 expression and the presence of metastasis at initial diagnosis (*P* = 0.039), and we found that four patients who had metastatic disease at the time of initial diagnosis were all ranked in the high-RPN2 group (**Table 1**). Among 31 patients who showed no metastasis at initial diagnosis, 12 developed lung metastasis during or after treatment, and the other 19 showed no metastasis for at least 3 years after treatment. Expression of RPN2 was significantly higher in the metastasis-positive group (*n* = 12, 2.04 ± 0.97) than in the group with no metastasis (*n* = 19, 1.42 ± 0.48) (**Figure 1e**). Although we found a close correlation between high-RPN2 expression and a poor response to neoadjuvant chemotherapy, it was not statistically significant (*P* = 0.063) (**Table 1**). We found no significant correlations between RPN2 expression and other factors such as patient gender, tumor site, or histological subtype (**Table 1**). These data suggested that higher expression of RPN2 in osteosarcoma might be associated with the metastatic phenotype and could be of novel prognostic value.

### RPN2 regulates drug response and invasiveness of osteosarcoma cells

**Figure 2:**
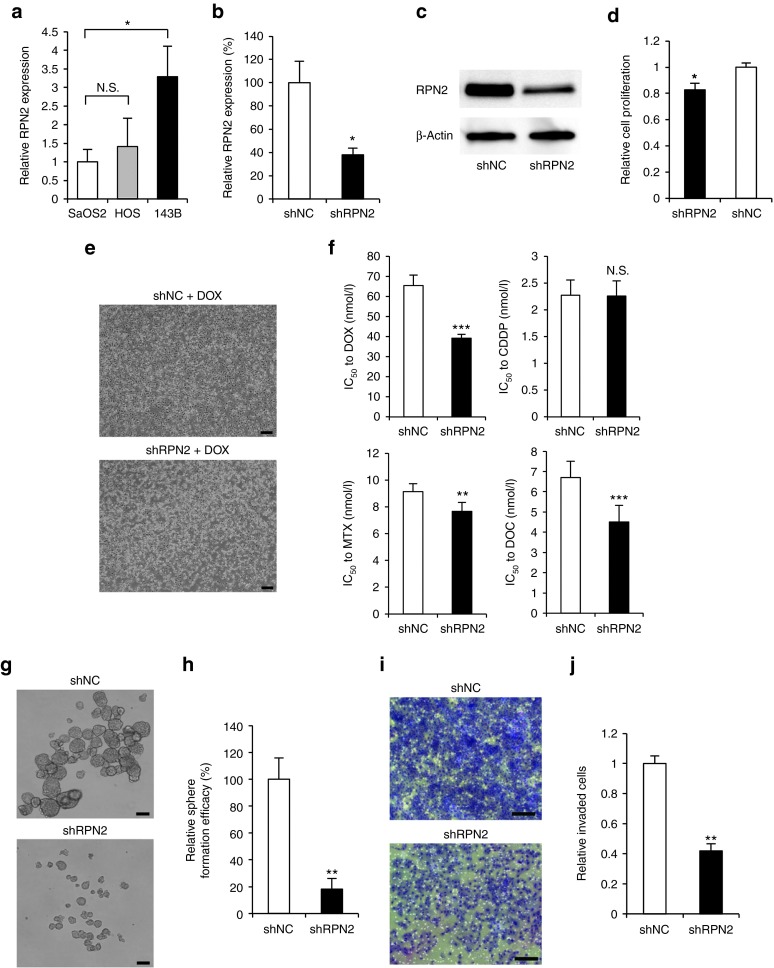
**RPN2 regulates malignant phenotypes of osteosarcoma cells**. (**a**) The relative expression levels of RPN2 mRNA in osteosarcoma cell lines. Data are presented as mean ± SD (*n* = 3 per group). N.S., not significant, *P < 0.05; Student's *t*-test. (**b**) Knockdown of RPN2 mRNA by shRNA, as confirmed using real-time RT-PCR. Data are presented as mean ± SD (*n* = 3 per group). *P < 0.05; Student's *t*-test. (**c**) Western blot analysis of RPN2 protein in 143B-shRPN2 and 143B-shNC cells. (**d**) Relative proliferation rates of 143B-shRPN2 and 143B-shNC cells on day 3. Data are presented as mean ± SD (*n* = 3 per group). *P < 0.05; Student's *t*-test. (**e**) Phase-contrast micrograph of 143B-shRPN2 and 143B-shNC cells in the presence of 400 nmol/l doxorubicin (DOX). Scale bar, 200 μm. (**f**) Drug sensitivity of 143B-shRPN2 and 143B-shNC cells in the presence of doxorubicin (DOX), cisplatin (CDDP), methotrexate (MTX), and docetaxel (DOC). Data are presented as mean ± SD (*n* = 6 per group). N.S., not significant, **P < 0.01, ***P < 0.001; Student's *t*-test. (**g**) Phase-contrast micrograph of the 143B-shRPN2 and 143B-shNC cells in a serum-free, growth factor-supplemented, anchorage-independent environment. Scale bar, 100 μm. (**h**) Relative sphere formation efficacy of 143B-shRPN2 and 143B-shNC cells observed in **g**. Spheroids with a diameter beyond 100 μm were counted. Data are presented as mean ± SD (*n* = 3 per group). **P < 0.01; Student's *t*-test. (**i**) Phase-contrast micrograph of the invaded cells of 143B-shRPN2 and 143B-shNC cells. (**j**) Relative invasiveness of 143B-shRPN2 and 143B-shNC cells observed in **i**. Data are presented as mean ± SD (*n* = 3 per group). **P < 0.01; Student's *t*-test.

To evaluate the functional effects of regulating RPN2 expression in osteosarcoma cells, we first confirmed the expression of RPN2 mRNA in several osteosarcoma cell lines. As a result, we found higher expression of RPN2 in 143B, a highly metastatic osteosarcoma cell line, than in SaOS2 or HOS, which are poorly metastatic osteosarcoma cell lines (**Figure 2a**). We then established stable clones of 143B expressing short hairpin RNA (shRNA) against RPN2 (143B-shRPN2) and control shRNA (143B-shNC). The reduced expression of RPN2 was confirmed by RT-PCR (**Figure 2b**), as well as by Western blot analysis (**Figure 2c**). Cell proliferation was slightly inhibited by RPN2 silencing (**Figure 2d**). After 48 hours of doxorubicin treatment, we found substantial cell death in 143B-shRPN2 relative to the control 143B-shNC (**Figure 2e**). We then tested the tumor cell responses to a wide range of drugs that have been used for treatment of osteosarcoma, and found that RPN2 silencing increased the sensitivity to doxorubicin, methotrexate, and docetaxel (**Figure 2f** and **Supplementary Figure S2**). In comparison with control 143B-shNC cells, 143B-shRPN2 formed fewer and smaller spheres in a serum-free, growth factor-supplemented, anchorage-independent environment (**Figure 2g**,**h**). Additionally, we analyzed the effect of RPN2 silencing on cell invasion, and found that 143B-shRPN2 cells were less invasive than 143B-shNC cells (**Figure 2i**,**j**).

### RPN2 expression in osteosarcoma cells is induced by doxorubicin treatment

**Figure 3:**
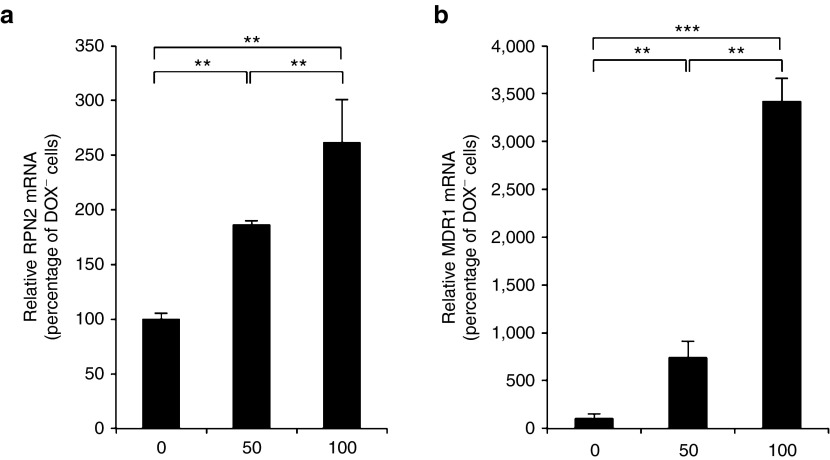
**Induction of RPN2 and MDR1 expression by doxorubicin treatment.** (**a**) RPN2 expression levels induced by doxorubicin treatment in 143B cells. The data shown are from 48 hours after doxorubicin treatment. Data are presented as mean ± SD (*n* = 3 per group). **P <0.01; Student's *t*-test. (**b**) MDR1 expression levels induced by doxorubicin treatment in 143B cells. The data shown are from 48 hours after doxorubicin treatment. Data are presented as mean ± SD (*n* = 3 per group). **P < 0.01, ***P < 0.001; Student's *t*-test.

Our previous investigation had shown that expression of RPN2 mRNA in docetaxel-sensitive breast cancer cells was markedly and dose-dependently induced by docetaxel. To confirm the effect of RPN2 mRNA expression in osteosarcoma cells by treatment with currently used drugs, we performed qRT-PCR for 143B cells after doxorubicin treatment. We found that expression of mRNA for both RPN2 and multidrug resistance gene 1 (MDR1) in 143B cells was markedly and dose-dependently induced by doxorubicin after 48 hours of treatment (**Figure 3a**,**b**). These data indicated that the cells surviving after doxorubicin treatment expressed a high amount the MDR1 and RPN2 gene products, suggesting that the development of drug resistance might correlate with induction of their expression in osteosarcoma cells.

### RPN2 silencing contributes to the inhibition of tumor growth and lung metastasis formation

**Figure 4:**
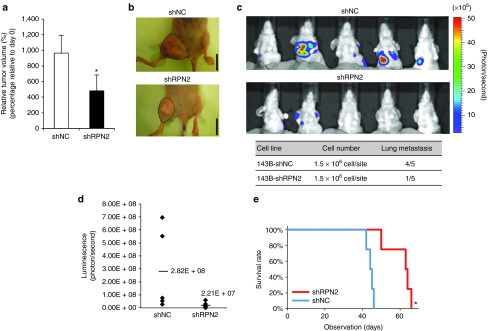
**Role of RPN2 in osteosarcoma primary tumor growth and metastasis.** (**a,b**) Tumors at the primary site of each group measured at 3 weeks after inoculation. The size of each tumor in mice was measured (**a**). Data are presented as mean ± SD (*n* = 5 per group). *P < 0.05; Student's *t*-test. The macroscopic appearances of 143B-shRPN2 and 143B-shNC tumors are shown (**b**). The tumor masses are outlined by a dotted line. Scale bars, 10 mm. (**c,d**) The lung metastases of each group measured on day 22 using an *in vivo* imaging system (**c**). The luminescence of the chest lesions in each group of mice was determined (**d**). (**e**) Survival curves for each group of mice by Kaplan–Meier analysis. Log-rank test was performed between the two groups (*P = 0.020).

To examine the role of RPN2 in primary tumor growth and metastasis, we transplanted 143B-shRPN2 and 143B-shNC cells into mice and evaluated the resulting tumor progression. 143B-shRPN2 (*n* = 5) and 143B-shNC (*n* = 5) were orthotopically implanted into the right proximal tibia of 4- to 6-week-old athymic nude mice at 1.5 × 10^6^ cells/mouse. The growth of the implanted tumors was measured once a week, and the presence of lung metastases was analyzed weekly by luciferase bioluminescence using an *in vivo* imaging system. We found that the primary tumor growth of 143B-shRPN2 was less than that of 143B-shNC (**Figure 4a**,**b**). After 3 weeks of orthotopic transplantation, there was significantly lesser lung metastasis in 143B-shRPN2-bearing mice than in 143B-shNC-bearing mice: four of five 143B-shRPN2-bearing mice exhibited lung metastases in comparison with only one of five 143B-shNC-bearing mice (**Figure 4c**). We evaluated the intensity of luminescence of chest lesions and identified apparently less signal intensity in 143B-shRPN2-bearing mice (**Figure 4d**). Presented differently, 143B-shRPN2-bearing mice had an 87% lower metastasis index than 143B-shNC-bearing mice (**Supplementary Figure S3**). All the mice were evaluated for survival, and 143B-shRPN2-bearing mice showed longer survival than 143B-shNC-bearing mice (log-rank test, *P* = 0.020) (**Figure 4e**), suggesting that decreased RPN2 expression provided a survival advantage on osteosarcoma-bearing mice.

## Discussion

An enormous body of research has been directed to overcome the lethal phenotypes of malignant neoplasms. Recent progress in targeted therapies has opened a new avenue in the treatment of sarcomas. However, little have been proven to be more effective than conventional therapies.^21,22^ Therefore, there is an urgent need to develop novel treatments for osteosarcoma. In this context, we have shown that RNA interference for RPN2 suppresses cell proliferation, sphere formation ability, and invasiveness, and increases the sensitivity of osteosarcoma cells to a wide range of chemotherapeutic drugs *in vitro*. Notably, RPN2 silencing inhibited tumor growth as well as lung metastasis formation, leading to a survival advantage of osteosarcoma-bearing mice. Furthermore, we found a close correlation between RPN2 expression and the clinicopathological features such as metastatic status and prognosis.

Using gene expression profiling of breast cancer biopsy samples between responders and nonresponders to docetaxel, Iwao-Koizumi *et al*. devised a diagnostic system that was able to predict the clinical response to docetaxel treatment, and identified molecular targets for therapy.^23^ As an extension of their report, we previously performed a study of RNAi-induced gene knockdown in docetaxel-resistant breast cancer cells, and identified the RPN2 gene, which is part of the N-oligosaccharyl transferase complex, as a new target for overcoming the drug resistance of breast cancer. Specifically, silencing of RPN2 reduced the glycosylation of the P-glycoprotein and decreased its membrane localization, thereby sensitizing cancer cells to docetaxel. A recent study by Kurashige *et al*. has shown that RPN2 expression is also able to predict the docetaxel response of esophageal squamous cell carcinoma. Silencing of RPN2 increased the sensitivity of esophageal cancer cells to docetaxel. However, the function and correlation of RPN2 expression with the clinical features of other malignancies, including mesenchymal neoplasms, remains to be elucidated. In this study of osteosarcoma cells, we demonstrated that silencing of RPN2 increased cell sensitivity to doxorubicin, methotrexate, and docetaxel. Doxorubicin and methotrexate are standard drugs for treatment of osteosarcoma, the former being especially effective.^24^ Since osteosarcoma patients who show a poor response to these drugs have a poor prognosis,^10,18^ silencing of RPN2 in osteosarcoma tissue would improve prognosis by sensitizing the cancer cells to these drugs. Furthermore, studies of second-line chemotherapy for osteosarcoma have made progress in recent years.^25^ In phase 2 trials with gemcitabine or docetaxel alone, up to 8% of patients with bone or soft tissue sarcomas showed objective responses.^26,27^ When gemcitabine was combined with docetaxel in a series of 10 patients with recurrent or progressive osteosarcoma, three patients showed partial responses and one showed stable disease.^28^ Since silencing of RPN2 could sensitize osteosarcoma to docetaxel, this approach might also be effective for patients with recurrent or progressive osteosarcoma. Collectively, the RPN2 gene may represent a novel target for RNAi therapeutics against a wide range of malignant neoplasms.

Our human study demonstrated that high expression of RPN2 in biopsy samples of osteosarcoma was significantly correlated with patient prognosis. Immunohistochemically, however, all specimens in this sample set were moderately to strongly positive for RPN2 protein. Therefore, we were unable to predict metastatic ability or prognosis on the basis of immunohistochemical staining for RPN2 protein. However, this result indicated that silencing of RPN2 may contribute to sensitization of osteosarcoma cells to chemotherapeutics in all patients. Among 16 high-RPN2 patients who showed a tumor response to neoadjuvant chemotherapy, 13 (81%) were poor responders. These data suggested that patients showing higher expression of RPN2 in osteosarcoma might tend to be poor responders to neoadjuvant chemotherapy, although the difference between these groups was not statistically significant. We considered that the correlation between high-RPN2 expression in biopsy samples and prognosis might have been due to the metastatic expression of osteosarcoma, since RPN2 expression was significantly correlated with clinical metastasis. Therefore, we analyzed the correlation between RPN2 expression and cell invasion, which is one of the important phenotypes associated with metastasis.

We found that RPN2 expression also regulates the invasiveness of osteosarcoma cells, representing a novel function of RPN2. Although the molecular mechanisms responsible for regulation of invasiveness via RPN2 protein are unclear, previous reports have demonstrated that N-linked glycosylation correlates with tumor cell invasion or metastatic phenotypes.^29,30,31,32,33,34,35^ N-glycosylation of integrins plays an important role in their biological functions.^31,32,33^ Integrins, cell surface transmembrane glycoproteins that function as adhesion receptors between cell and extracellular matrix and link matrix proteins to the cytoskeleton, play an important role in cytoskeletal organization and in the transduction of intracellular signals, regulating various processes such as proliferation, differentiation, apoptosis, and cell migration.^32^ Reportedly, in comparison with the non-metastatic WM35 melanoma cell line, α1 and β3 subunits, expressed by the metastatic A375 melanoma cell line, carry β1,6 GlcNAc branched structures, suggesting that these cancer-associated glycan chains may modulate tumor cell adhesion by affecting the ligand-binding properties of α1β3 integrin.^35^ Another report has shown that N-glycosylation is essential for the function of integrin α5β1, and that any alteration in the expression of N-glycans in α5β1 integrin would contribute to the adhesive and metastatic properties of tumors. When NIH3T3 cells were transformed with the oncogenic *Ras* gene, cell spreading on fibronectin was greatly enhanced due to an increase in β1,6 GlcNAc branched tri- and tetra-antennary oligosaccharides in α5β1 integrin.^29^ Indeed, α5β1 integrin is related to tumor cell invasion and metastatic potential in osteosarcoma cells.^36^ Therefore, inhibition of osteosarcoma cell invasion was caused by RPN2 silencing via alteration of N-glycosylation status of this molecule. Furthermore, a novel function of RPN2-mediated tumor cell malignancy was recently reported. RPN2 silencing resulted in reduced CD63 glycosylation and deregulated localization in tumor cells, which regulates drug resistance and tumor cell invasion. Collectively, the glycosylation status of several molecules associated with tumor cell invasion may be regulated by RPN2 expression.^37^ Moreover, considering the regulation of sphere formation ability of osteosarcoma cells, RPN2 might be correlated with cancer stem cell properties of osteosarcoma, which was also indicated in breast cancer cells.^37^ Since the direct interaction of these phenotypes with RPN2 in osteosarcoma has not been elucidated, further study is needed to clarify the molecular mechanisms underlying the tumor-suppressive function by RPN2 silencing.

In this study, we found that RPN2 silencing contributed to inhibition of tumor growth and lung metastasis formation *in vivo*. Previously, we showed that atelocollagen-mediated RPN2 small interfering RNA (siRNA) delivery markedly reduced tumor growth in murine breast cancer models.^20^ In recent years, RNA interference (RNAi) therapeutics, most notably with lipid nanoparticle-based delivery systems, have advanced to the human clinical trial stage.^38,39,40,41^ One of the most advanced trials included a study from the United States in 2010 demonstrating that systemic administration of siRNA via targeted nanoparticles reduced the levels of both specific mRNA (the M2 subunit of ribonucleotide reductase; RRM2) and protein (RRM2) in melanoma biopsy specimens, representing the first human phase 1 clinical trial for patients with solid cancers.^38^ Our preclinical trial of RPN2 silencing suggests that it would be worth evaluating the efficacy of siRNA administration, which is our next goal. In fact, a clinical phase 1 study of siRNA targeting RPN2 is now in the preliminary stage at our institution, and it is anticipated that this will yield novel information on treatments for solid cancers.

Our study had several limitations that warrant consideration. First, the number of patients in our clinical cohort was relatively small. For this reason, we were unable to draw any clear conclusions about the correlation between RPN2 expression in biopsy samples and tumor response to neoadjuvant chemotherapy, while most patients in the high-RPN2 group were poor responders. A larger series with more patients will therefore be needed to validate these results, and for this purpose we are continuing to collect biopsy specimens from osteosarcoma patients. Second, as RPN2 regulates the membrane localization of P-glycoprotein via N-linked glycosylation, the glycosylation status of the invasion-related molecules might also be affected, which we plan to elucidate. Third, the molecular mechanisms underlying RPN2 upregulation in highly malignant cells or during a drug response have not been elucidated. Therefore, we plan to further investigate the molecular mechanisms underlying RPN2 upregulation and the interactions of N-linked glycosylation with invasion-related molecules with RPN2 in osteosarcoma cells.

In summary, we have shown that the RPN2 gene is moderately to strongly expressed in all osteosarcomas, and that higher RPN2 expression is significantly correlated with clinical metastasis and poor patient survival. Furthermore, silencing of RPN2 contributes to reduction of cell proliferation, sphere formation, and invasiveness, and sensitizes osteosarcoma cells to standard chemotherapeutic regimens, thus providing a survival advantage on osteosarcoma-bearing mice. These data indicate that the RPN2 gene may represent a novel target for RNAi therapeutics against osteosarcoma.

## Materials and Methods

*Cells and cell culture.* Three human osteosarcoma cell lines (SaOS2, HOS, and 143B) were purchased from the American Type Culture Collection (ATCC, Manassas, VA), and maintained in Dulbecco's modified Eagle's medium (DMEM) (Life Technologies, Carlsbad, CA). All media were supplemented with 10% heat-inactivated fetal bovine serum (Life Technologies), penicillin (100 U/ml), and streptomycin (100 μg/ml), and the cells were maintained under 5% CO_2_ in a humidified incubator at 37 °C.

*Lentiviral shRNA transduction.* Cell lines stably expressing RPN2 shRNA or control non-target shRNA were established using a vector-based shRNA technique (**Supplementary Figure S1**). Human RPN2 shRNA targets 5′-GGAGGAGATTGAGGACCTTGT-3′ (shRPN2-site1), 5′-GCCACTTTGAAGAACCCAATC-3′ (shRPN2-site2), 5′-TCCAGATTGTAGTTATACTTC-3′ (shRPN2-UTR), and control shRNA targets 5′-GAAATGTACTGCGCGTGGAGAC-3′. Briefly, each fragment was subcloned into pGreenPuro (System Biosciences, Tokyo, Japan). Recombinant lentiviruses were produced in accordance with the manufacturer's instructions. In knockdown experiments, 143B cells were infected with recombinant lentiviruses expressing control shRNA (shNC) or shRNA against RPN2 (shRPN2).^37^

*RNA isolation and qRT-PCR.* We purified total RNA from tumor cells and tissues using the RNeasy Mini Kit and RNase-Free DNase Set (QIAGEN, Tokyo, Japan). For qPCR of mRNAs, cDNA was synthesized using a High-Capacity cDNA Reverse Transcription Kit (Life Technologies). For each qPCR, equal amounts of cDNA were mixed with Platinum SYBR Green qPCR SuperMix (Life Technologies) and the specific primers (**Supplementary Table S1**). We normalized gene expression levels to β-actin or *GAPDH*.

*Western blotting.* Western blotting was performed as described previously.^20^ The membranes were blotted with a rabbit polyclonal antibody against human RPN2 antigen (1:100 dilution, H-300, Santa Cruz Biotechnology, Santa Cruz, CA), or with a monoclonal antibody against β-actin (1:2,000, AC-15, Sigma, St Louis, MO). Signals were visualized with an enhanced chemiluminescence system (ECL Detection System; Amersham Pharmacia Biotech Piscataway, NJ).

*Cell proliferation and cytotoxicity assays.* The cell proliferation rates and cell viability were determined using the TetraColor ONE Cell Proliferation Assay (Seikagaku, Tokyo, Japan) or Cell proliferation kit 8 (Dojindo, Kumamoto, Japan), according to the manufacturer's instructions. Cells growing in the logarithmic phase were seeded in 96-well plates (5 × 10^3^/well), allowed to attach overnight, and then treated with varying doses of doxorubicin (Sigma-Aldrich, St. Louis, MO), cisplatin (Enzo Life Sciences, Farmingdale, NY), methotrexate (Sigma-Aldrich), and docetaxel (Sigma-Aldrich) for 72 hours. Triplicate wells were used for each treatment group. The absorbance was measured at 450 nm with a reference wavelength at 650 nm using EnVision (Perkin-Elmer, Waltham, MA). The relative number of viable cells was expressed as a percentage of the total number.

*Sphere formation.* Osteosarcoma cells were plated at 100 cells/well in 300 μl of serum-free DMEM/F12 medium (Life Technologies) supplemented with 20 ng/ml human recombinant EGF (Sigma-Aldrich), 10 ng/ml human recombinant bFGF (Life Technologies), 4 μg/ml insulin (Sigma-Aldrich), B27 (1:50; Invitrogen), 500 units/ml penicillin (Life Technologies), and 500 μg/ml streptomycin (Life Technologies). The cells were cultured in suspension in 24-well ultra-low attachment plates (Corning, Corning, NY), and replenished with 30 μl of new medium every second day. The spheres were counted on day 5 in triplicate wells. Cell culture was maintained at 37 °C in a 5% CO_2_ humidified incubator.

*Invasion assays.* Invasion assays were performed using 24-well BD BioCoat Invasion Chambers with Matrigel (Becton-Dickinson, Tokyo, Japan). A total of 1 × 10^5^ cells were suspended in 500 μl DMEM medium without fetal bovine serum and added to the upper chamber. DMEM medium with 10% fetal bovine serum was added to the lower chamber. After incubation for 24 hours, the cells on the upper surface of the filter were completely removed by wiping with cotton swabs. The filters were fixed in methanol and stained with 1% toluidine blue in 1% sodium tetraborate (Sysmex, Kobe, Japan). The filters were then mounted onto slides, and the cells on the lower surfaces were counted.

*Animal experiments.* Animal experiments in this study were performed in compliance with the guidelines of the Institute for Laboratory Animal Research, National Cancer Center Research Institute. Four- to six-week-old female Balb/c athymic nude mice (CLEA Japan, Tokyo, Japan) were anesthetized by exposure to 3% isoflurane for injections and *in vivo* imaging. On day 0, the mice were anesthetized with 3% isoflurane, and the right leg was disinfected with 70% ethanol. The cells were aspirated into a 1-ml tuberculin syringe fitted with a 27-G needle. The needle was inserted through the cortex of the anterior tuberosity of the tibia with a rotating movement to avoid cortical fracture. Once the bone was traversed, the needle was inserted further to fracture the posterior cortex of the tibia. A 100 μl volume of solution containing 1.5 × 10^6^ cells of 143B-shRPN2 (*n* = 5) or 143B-shNC (*n* = 5) cells was injected while slowly removing the needle. Tumor size was monitored by measuring tumor length and width using calipers. The volumes of 143B-shRPN2 or 143B-shNC tumors were calculated using the formula: (*L* + *W*) × *L* × *W* × 0.2618, where *L* is the length and *W* is the width of each tumor as reported previously.^42^ To evaluate lung metastases, mice were injected with d-luciferin (150 mg/kg, Promega, Tokyo, Japan) by intraperitoneal injection. After 10 minutes, the photons from the firefly luciferase were counted using the *in vivo* imaging system (Xenogen, Alameda, CA) according to the manufacturer's instructions. Data were analyzed using LIVINGIMAGE 4.3.1 software (Xenogen).

*Human samples.* The osteosarcoma tissue samples were obtained from diagnostic incisional biopsies of primary osteosarcoma sites before the start of neoadjuvant chemotherapy at the National Cancer Center Hospital, Tokyo, between 1997 and 2010. Patients older than 40 years of age or patients who had primary tumors located outside the extremities were excluded. Each fresh tumor sample was cut into two pieces; one piece was immediately cryopreserved in liquid nitrogen, and the other piece was fixed in formalin. The diagnosis of osteosarcoma and the histological subtypes were determined by certified pathologists. The response to chemotherapy was classified as good if the tumor necrosis was 90% or greater. All patients provided written informed consent authorizing the collection and use of their samples for research purposes. The study protocol for obtaining clinical information and collecting samples was approved by the Institutional Review Board of the National Cancer Center of Japan.

*Immunohistochemistry.* To stain RPN2, we prepared slides from clinical samples of osteosarcoma. Endogenous peroxidase was quenched with 1% H_2_O_2_ (30 minutes). The slides were heated for antigen retrieval in 10 mmol/l sodium citrate (pH 6.0). Subsequently, we incubated the slides with RPN2-specific antibody (1:100 dilution, H-300, Santa Cruz Biotechnology) and isotype-matched control antibodies overnight at 4 °C. Immunodetection was performed using ImmPRESS peroxidase polymer detection reagents (Vector Laboratories, Burlingame, CA) and the Metal-Enhanced DAB Substrate Kit (Thermo Fisher Scientific, Yokohama, Japan) in accordance with the manufacturer's directions. Sections were counterstained with hematoxylin for contrast.

*Statistical analysis.* All statistical analyses were performed using SPSS Statistics Version 21 software (IBM SPSS, Tokyo, Japan). Student's *t*-test or Welch's *t*-test, was used to determine the significance of any differences between experimental groups. Differences in RPN2 expression among different clinicopathological data were analyzed using the chi-squared (*χ*^2^) test. We performed ROC curve analysis using the SPSS software, and the optimal cutoff points for the expression levels of RPN2 were determined by the Youden index, *i.e*., *J* = max (sensitivity + specificity – 1).^43^ The Kaplan–Meier method and the log-rank test were used to compare the survival of patients. We defined the survival period as the time from diagnosis until death, whereas living patients were censored at the time of their last follow-up. For all the analyses, we considered a *P* value of 0.05 or less to be significant.

**SUPPLEMENTARY MATERIAL** **Figure S1.** Diagram of the lentiviral vector utilized in the present study. **Figure S2.** Phase-contrast micrograph of 143B-shRPN2 and 143B-shNC cells in the presence of 50 nmol/l methotrexate (**a**), 10 μmol/l cisplatin (**b**), and 10 nmol/l docetaxel (**c**). **Figure S3.** Metastasis index (= intensity of lung metastasis luminescence/primary tumor size) in 143B-shRPN2-bearing mice or 143B-shNC-bearing mice at 3 weeks after orthotopic implantation. **Table S1.** The sequences of primers used for real-time RT-PCR analysis.

## Figures and Tables

**Table 1:**
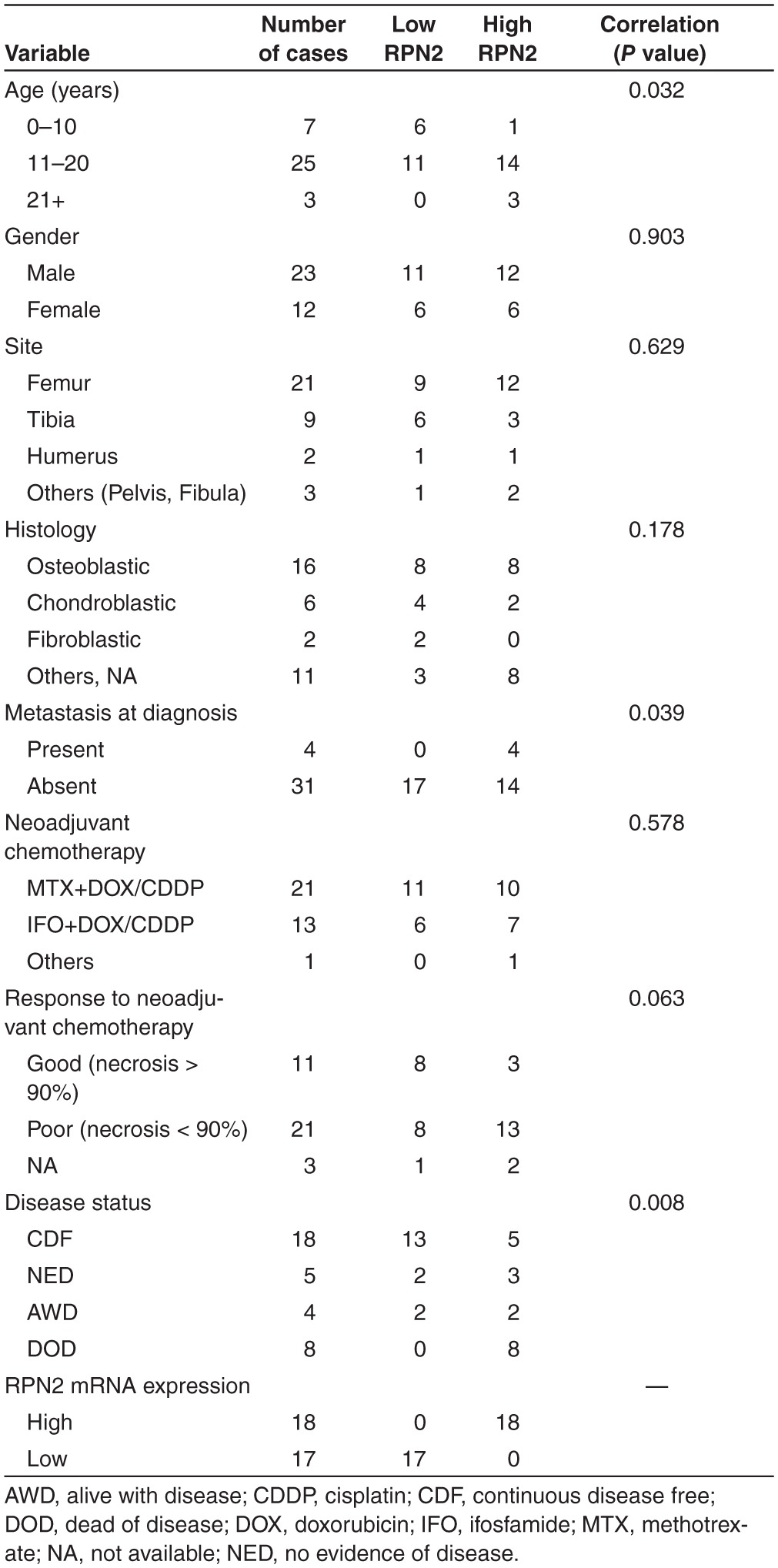
Clinicopathological correlation of RPN2 expression in osteosarcoma

## Supplementary Material

Supplementary Figure S1 Diagram of the lentiviral vector utilized in the present study.

Supplementary Figure S2 Phase-contrast micrograph of 143B-shRPN2 and 143B-shNC cells in the presence of 50 nmol/l methotrexate.

Supplementary Figure S3 Metastasis index (= intensity of lung metastasis luminescence/primary tumor size) in 143B-shRPN2-bearing mice or 143B-shNC-bearing mice at 3 weeks after orthotopic implantation.

Supplementary Table S1 The sequences of primers used for real-time RT-PCR analysis.
