# Supplementary figures and images for: RPN2 Gene Confers Osteosarcoma Cell Malignant Phenotypes and Determines Clinical Prognosis
Source: Mol Ther Nucleic Acids. 2014 Sep 2;3(9):e189–. doi: 10.1038/mtna.2014.35 (PMC4222647; doi:10.1038/mtna.2014.35)

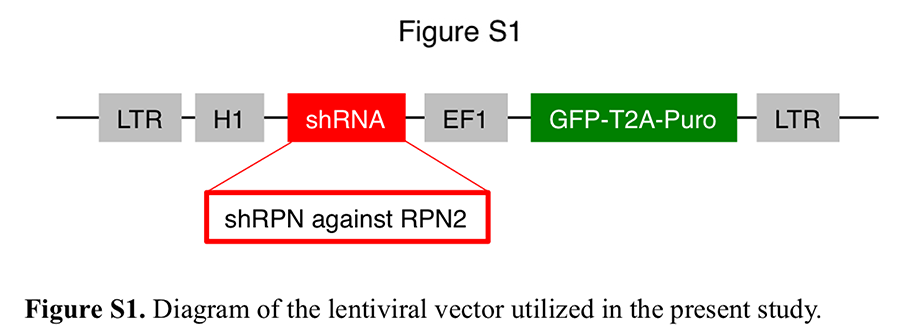

Supplement: Supplementary Figure S1 — Diagram of the lentiviral vector utilized in the present study. [file mtna201435x1.tiff]

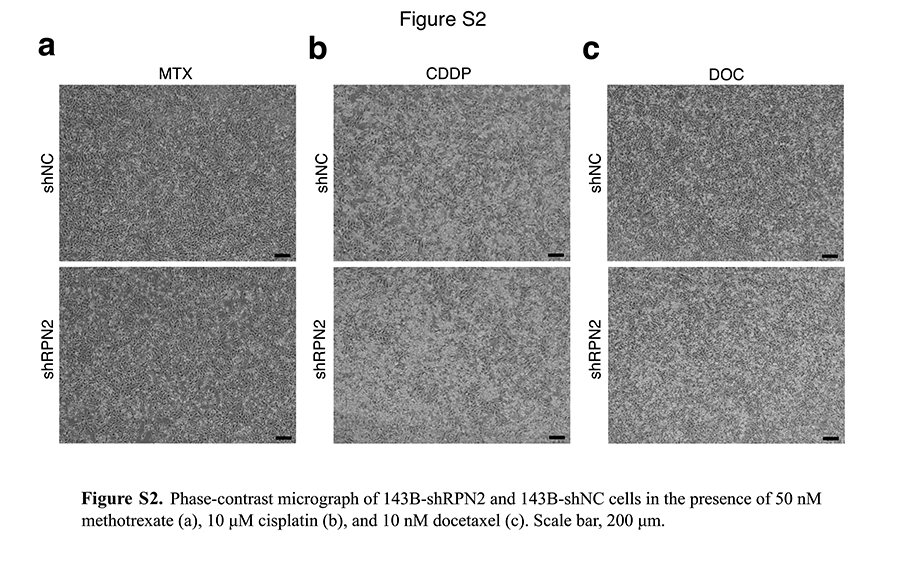

Supplement: Supplementary Figure S2 — Phase-contrast micrograph of 143B-shRPN2 and 143B-shNC cells in the presence of 50 nmol/l methotrexate. [file mtna201435x2.tiff]

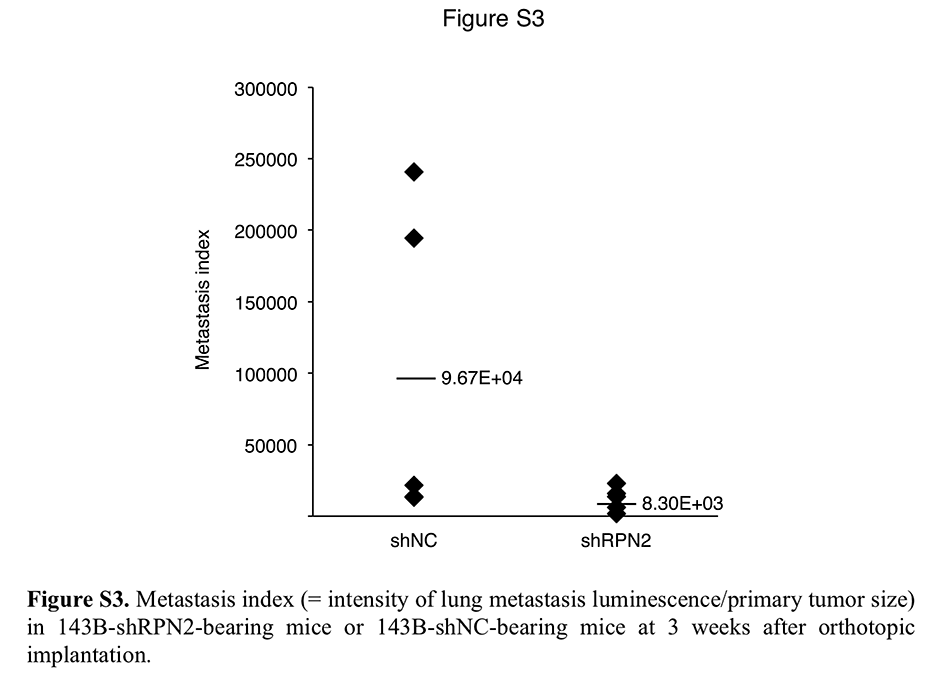

Supplement: Supplementary Figure S3 — Metastasis index (= intensity of lung metastasis luminescence/primary tumor size) in 143B-shRPN2-bearing mice or 143B-shNC-bearing mice at 3 weeks after orthotopic implantation. [file mtna201435x3.tiff]

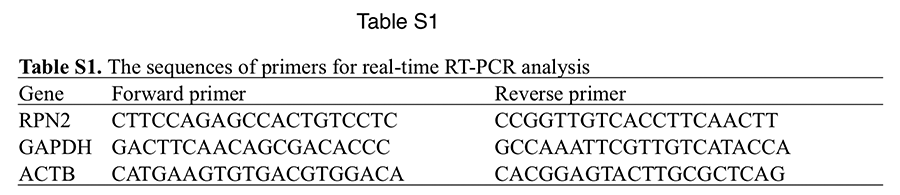

Supplement: Supplementary Table S1 — The sequences of primers used for real-time RT-PCR analysis. [file mtna201435x4.tiff]
